# Supplementary material for: The NADPH Oxidase A of Verticillium dahliae Is Essential for Pathogenicity, Normal Development, and Stress Tolerance, and It Interacts with Yap1 to Regulate Redox Homeostasis
Source: J Fungi (Basel). 2021 Sep 9;7(9):740. doi: 10.3390/jof7090740 (PMC8468606; doi:10.3390/jof7090740)
Supplement: Supplementary file 1 [file jof-07-00740-s001.zip › Supplementary_Figures.pdf]

Supplementary Figures

The NADPH Oxidase A of *Verticillium dahliae* is Essential for Pathogenicity, Normal Development, and Stress Tolerance, and it Interacts with Yap1 to Regulate Redox Homeostasis

Vasileios Vangalis[1], Ioannis A. Papaioannou[2,*], Emmanouil A. Markakis[3], Michael Knop[2,4] and Milton A. Typas[1,*]

[1] Department of Genetics & Biotechnology, Faculty of Biology, National and Kapodistrian University of Athens, 15784, Athens, Greece; vasvagg@biol.uoa.gr (V.V.); matypas@biol.uoa.gr (M.A.T)
[2] Center for Molecular Biology of Heidelberg University (ZMBH), 69120, Heidelberg, Germany; i.papaioannou@zmbh.uni-heidelberg.de (I.A.P.); m.knop@zmbh.uni-heidelberg.de (M.K.)
[3] Laboratory of Mycology, Department of Viticulture, Vegetable Crops, Floriculture and Plant Protection, Institute of Olive Tree, Subtropical Crops and Viticulture, N.AG.RE.F., Hellenic Agricultural Organization - DIMITRA, 71307, Heraklion, Crete, Greece; markakis@elgo.iosv.gr (E.A.M.)
[4] German Cancer Research Center (DKFZ), DKFZ-ZMBH Alliance, 69120, Heidelberg, Germany
* Correspondence: i.papaioannou@zmbh.uni-heidelberg.de (I.A.P.); matypas@biol.uoa.gr (M.A.T.)

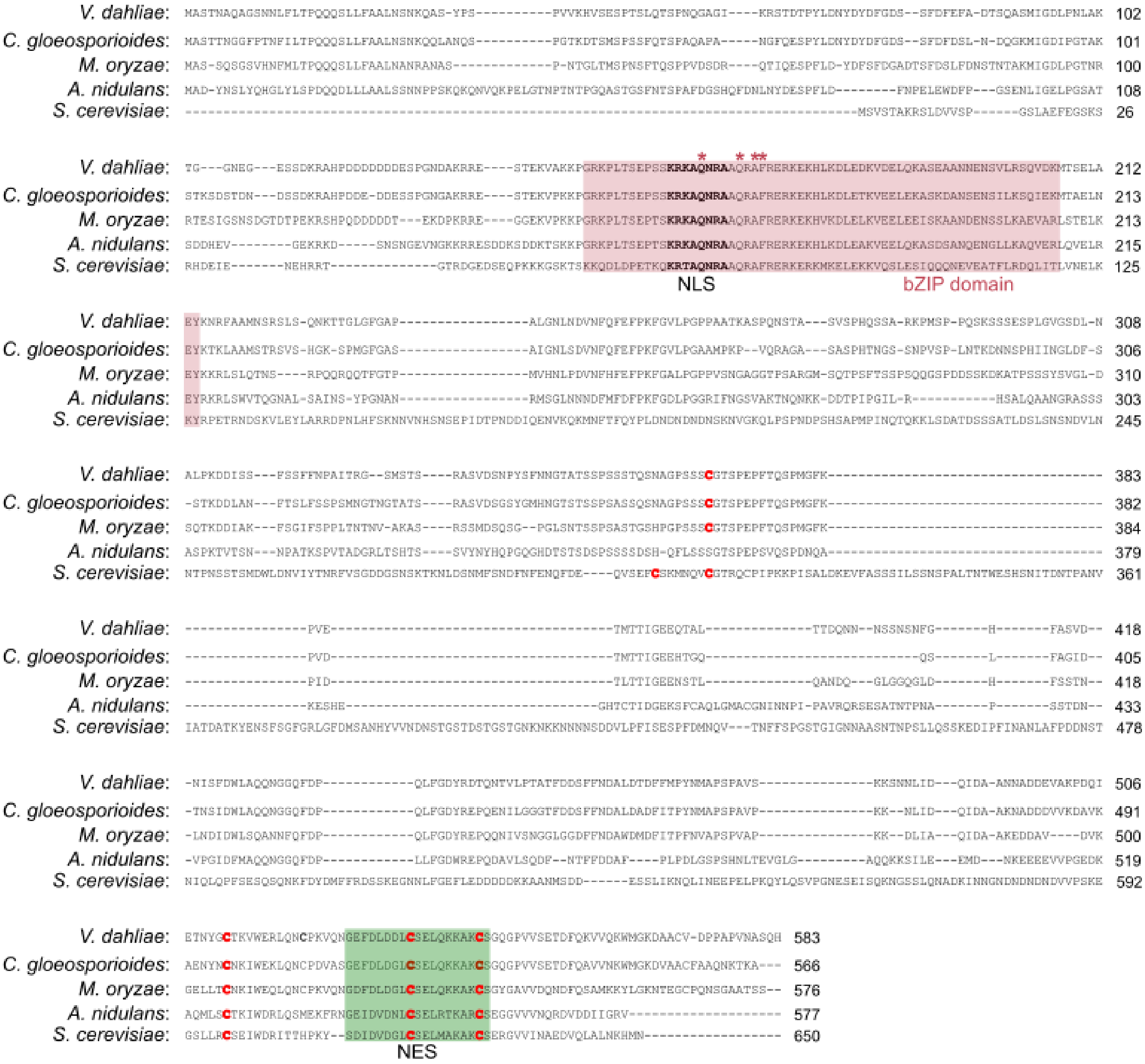

Figure S1. Sequence alignment of Yap1 homologs of *V. dahliae* (VDAG_01588) and other fungi (*Colletotrichum gloeosporioides*, CGLO_14059; *Magnaporthe oryzae*, MGG_12814; *Aspergillus nidulans*, AN7513; *Saccharomyces cerevisiae*, YML007W). The conserved bZIP domain is indicated by a purple box (asterisks indicate highly conserved characteristic residues of this domain). The nuclear localization sequence (NLS) is highlighted in bold. The green box indicates the nuclear export sequence (NES) of Yap1 homologs. Conserved catalytic cysteine residues are highlighted in bold red.

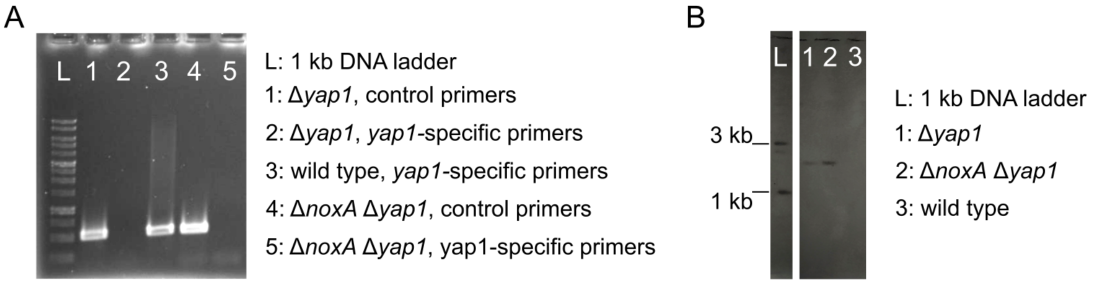

Figure S2. Validation of the *yap1* knockout mutants. (**A**) PCR validation of *yap1* deletion using *yap1*-specific primers (i.e., Vdap1F/R, Table S3, Supplementary Materials). Primers ctrlF/R (Table S3, Supplementary Materials) were used in control reactions for testing template quality. (**B**) Validation of mutants by Southern blotting after digestion of genomic DNA with *Xho*I, using a probe for the *neo*^{R} cassette.

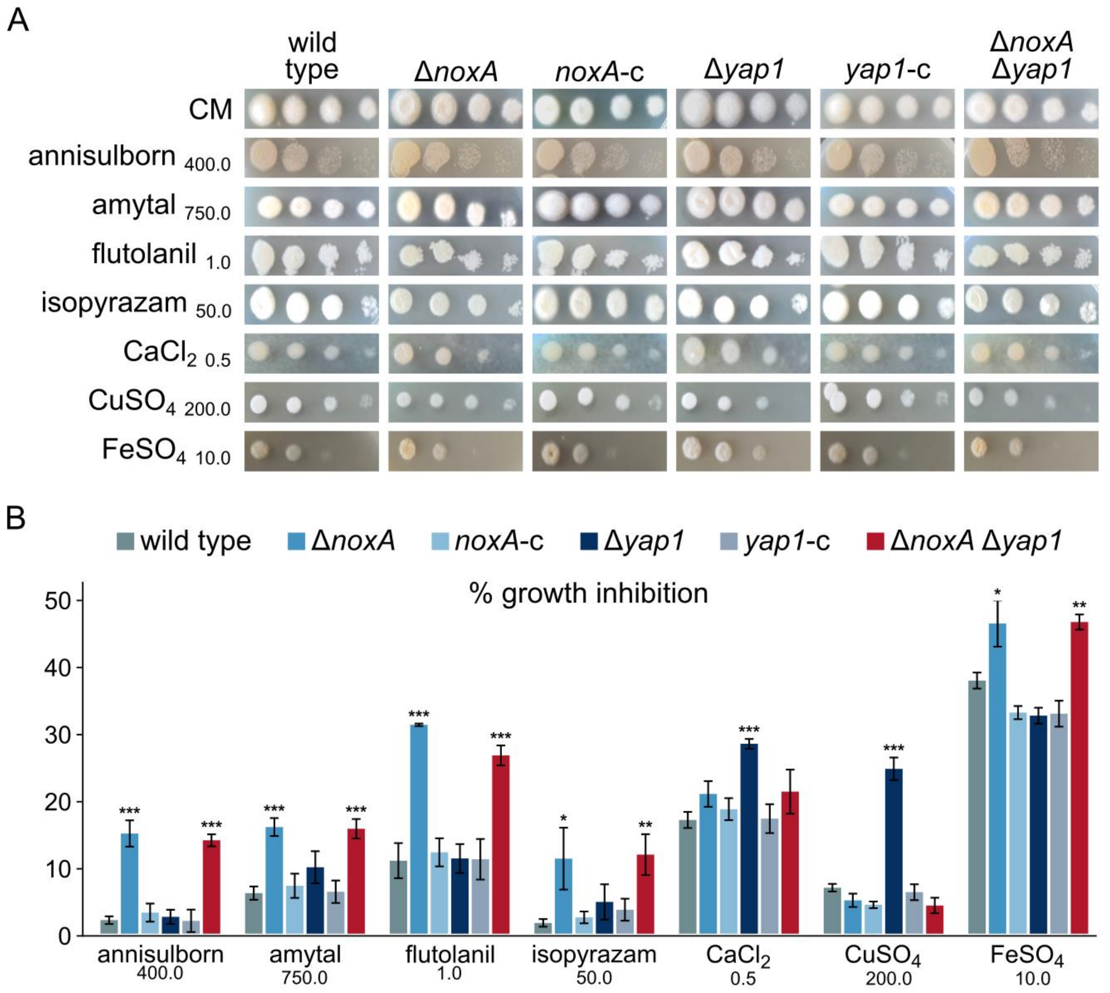

Figure S3. Effects of antifungal agents that target the respiratory chain, and trace elements on *V. dahliae* deletion mutants Δ*noxA* and Δ*yap1*. (**A**) Effects of annisulborn, sodium amytal, flutolanil, isopyrazam, $CaCl_2$, $CuSO_4$, and $FeSO_4$ on germination of strains (spot assays; growth for 3 days). (**B**) Relative growth inhibition of colonies caused by the same substances. Bars: SD. Statistical significance of differences from the wild type was tested by Student's *t*-tests (* $p \leq 0.05$, ** $p \leq 0.01$, *** $p \leq 0.001$). All concentrations are expressed in μg/mL, except for flutolanil, $FeSO_4$ (mM), and $CaCl_2$ (M). All experiments were performed in triplicate.
