# Supplementary material for: The NADPH Oxidase A of Verticillium dahliae Is Essential for Pathogenicity, Normal Development, and Stress Tolerance, and It Interacts with Yap1 to Regulate Redox Homeostasis
Source: J Fungi (Basel). 2021 Sep 9;7(9):740. doi: 10.3390/jof7090740 (PMC8468606; doi:10.3390/jof7090740)
Supplement: Supplementary file 1 [file jof-07-00740-s001.zip › Table_S1_v1.pdf]

Table S1: *Verticillium dahliae* strains constructed and used in this study.

strain	background	genotype	constructed using plasmid(s)
123V	-	wild type	–
123V-ΔnoxA	123V	Δ*noxA*::*hph*	pOSCAR-noxA
123V-noxA-c	123V-ΔnoxA	Δ*noxA*::*hph*, *noxA*, *neo*^{R}	pOSCAR-noxA, noxA, pSD1
123V-Δyap1	123V	Δ*yap1*::*neo*^{R}	pOSCAR-yap1
123V-yap1-c	123V-Δyap1	Δ*yap1*::*neo*^{R}, *yap1*, *hph*	pOSCAR-yap1, yap1, pUCATPH
123V-ΔnoxA Δyap1	123V-ΔnoxA	Δ*noxA*::*hph,* Δ*yap1*::*neo*^{R}	pOSCAR-noxA, pOSCAR-yap1
Ls.17 H1-sgfp	Ls.17	*nit*M, *NcH1-sgfp, hph*	pMF357
