# Supplementary material for: The NADPH Oxidase A of Verticillium dahliae Is Essential for Pathogenicity, Normal Development, and Stress Tolerance, and It Interacts with Yap1 to Regulate Redox Homeostasis
Source: J Fungi (Basel). 2021 Sep 9;7(9):740. doi: 10.3390/jof7090740 (PMC8468606; doi:10.3390/jof7090740)
Supplement: Supplementary file 1 [file jof-07-00740-s001.zip › Table_S2_v1.pdf]

Table S2: List of plasmids constructed and used in this study.

plasmid	backbone	description	source
pUCATPH	-	*hph*	[49]
pSD1	pBluescript II	P_{gpda}, P_{trpc}, *neo*^{R}	[47]
pOSCAR	pPZP-RCS2	*A. tumefaciens* binary vector	[48]
pOSCAR-yap1	pOSCAR	5'H_{yap1}-*neo*^{R}-3'H_{yap1} (H: 2.0 kb-long homology arms)	This study
