# Supplementary material for: The NADPH Oxidase A of Verticillium dahliae Is Essential for Pathogenicity, Normal Development, and Stress Tolerance, and It Interacts with Yap1 to Regulate Redox Homeostasis
Source: J Fungi (Basel). 2021 Sep 9;7(9):740. doi: 10.3390/jof7090740 (PMC8468606; doi:10.3390/jof7090740)
Supplement: Supplementary file 1 [file jof-07-00740-s001.zip › Table_S3_v1.pdf]

Table S3: List of DNA oligonucleotides used in this study.

oligo name	sequence (5' to 3')	template (strain or plasmid)	construction/ validation/expression of plasmid/strain/gene
	***yap1* deletion**		
5flap1F	GAAACGACAATCTGATCCAAGCTCAAGCTAGAGACATCGGGATTAAGCTG	123V	pOSCAR-yap1
5flap1R	CAATATCAGTTAACGTCGCTTGTTGGAGTTGAGAGC	123V	pOSCAR-yap1
ap1genF	CTCAACTCCAACAAGCGACGTTAACTGATATTGAAGGAGCAC	pSD1	pOSCAR-yap1
ap1genR	CTCAATGCTGGCTGGAACCCAGGGCTGGTGACGG	pSD1	pOSCAR-yap1
3flap1F	CACCAGCCCTGGGTTCCAGCCAGCATTGAGGAG	123V	pOSCAR-yap1
3flap1R	GCCTGCAGGTCGCGAGCGATCGCGGTACACAAGAAGTACGAGCTTGC	123V	pOSCAR-yap1
Vdap1F	CAAGCGGCAGCGAACATC	123V, 123V-Δyap1, 123V-ΔnoxA Δyap1, 123V-yap1-c	123V-Δyap1, 123V-ΔnoxA Δyap1, 123V-yap1-c
Vdap1R	GCGGGGTCCTCTTTGGTG	123V, 123V-Δyap1, 123V-ΔnoxA Δyap1, 123V-yap1-c	123V-Δyap1, 123V-ΔnoxA Δyap1, 123V-yap1-c
ctrlF	GCGCAGTAGGCCAGATCAGG	123V-Δyap1, 123V-ΔnoxA Δyap1	123V-Δyap1, 123V-ΔnoxA Δyap1, 123V-yap1-c
ctrlR	GTCCCGTGCTCGTTTGGTG	123V-Δyap1, 123V-ΔnoxA Δyap1	123V-Δyap1, 123V-ΔnoxA Δyap1, 123V-yap1-c
5flap1F	GAAACGACAATCTGATCCAAGCTCAAGCTAGAGACATCGGGATTAAGCTG	123V	123V-yap1-c
3flap1R	GCCTGCAGGTCGCGAGCGATCGCGGTACACAAGAAGTACGAGCTTGC	123V	123V-yap1-c
	**qPCR analysis**		
Vdtub-q-F	AGCTCACCCAGCAGATGTTC	123V, 123V-ΔnoxA, 123V-Δyap1, 123V-ΔnoxA Δyap1	β-tubulin (VDAG_10074)
Vdtub-q-R	TCGACCTCCTTCATGGCAAC	123V, 123V-ΔnoxA, 123V-Δyap1, 123V-ΔnoxA Δyap1	β-tubulin (VDAG_10074)
Vdcat1-q-F	TGGCTCCGTATTGCTAGC	123V, 123V-ΔnoxA, 123V-Δyap1, 123V-ΔnoxA Δyap1	*cat1* (VDAG_03661)
Vdcat1-q-R	CTTGCGTGAACAACTCTCTC	123V, 123V-ΔnoxA, 123V-Δyap1, 123V-ΔnoxA Δyap1	*cat1* (VDAG_03661)
Vdsod1-q-F	TGACCTCGGCAACATCAAG	123V, 123V-ΔnoxA, 123V-Δyap1, 123V-ΔnoxA Δyap1	*sod1* (VDAG_02630)
Vdsod1-q-R	CGACGGTACGGCCAATAAC	123V, 123V-ΔnoxA, 123V-Δyap1, 123V-ΔnoxA Δyap1	*sod1* (VDAG_02630)
Vdglr1-q-F	CATTCCCTCGGTCGTCTTC	123V, 123V-ΔnoxA, 123V-Δyap1, 123V-ΔnoxA Δyap1	*glr1* (VDAG_07524)
Vdglr1-q-R	GCGGTGAACTTGGTCTTGTAC	123V, 123V-ΔnoxA, 123V-Δyap1, 123V-ΔnoxA Δyap1	*glr1* (VDAG_07524)
Vdfus3-q-F	CATTCAGGATGTCGTTGGAG	123V, 123V-ΔnoxA, 123V-Δyap1, 123V-ΔnoxA Δyap1	*fus3* (VDAG_09461)
Vdfus3-q-R	GGTCGAAAGGAGTGATCTTC	123V, 123V-ΔnoxA, 123V-Δyap1, 123V-ΔnoxA Δyap1	*fus3* (VDAG_09461)
Vdslt2-q-F	CACTTCCAGTCCTTCATCTACC	123V, 123V-ΔnoxA, 123V-Δyap1, 123V-ΔnoxA Δyap1	*slt2* (VDAG_02584)
Vdslt2-q-R	GGCATTGACCAGCAAGTTTC	123V, 123V-ΔnoxA, 123V-Δyap1, 123V-ΔnoxA Δyap1	*slt2* (VDAG_02584)

Vdhog1-q-F	GACCACGTCAACCAGTTC	*hog1* (VDAG_08982)
Vdhog1-q-R	CAAGCATCTTCTCTAGCAGGTC	
Vdnox1-q-F	CTCCTCTCCTCCCTCGAATC	*nox1* (VDAG_06812)
Vdnox1-q-R	CTGGGTTGTGTCCATGTCG	
Vdyap1-q-F	CCAACAAGCAAGCGTCTTATC	*yap1* (VDAG_01588)
Vdyap1-q-R	GAGTCGCCAAAGTCATAGTCG	
